# Cross-Country Ski Skating Style Sub-Technique Detection and Skiing Characteristic Analysis on Snow Using High-Precision GNSS

**DOI:** 10.3390/s24186073

**Published:** 2024-09-19

**Authors:** Shunya Uda, Naoto Miyamoto, Kiyoshi Hirose, Hiroshi Nakano, Thomas Stöggl, Vesa Linnamo, Stefan Lindinger, Masaki Takeda

**Affiliations:** 1 Graduate School of Health and Sports Science, Doshisha University, 1-3 Tatara Miyakodani, Kyotanabe, Kyoto 610-0394, Japan; cyhj0007@mail4.doshisha.ac.jp (S.U.); cyhk0003@mail4.doshisha.ac.jp (H.N.); 2 Research Center for Sports Sensing, Doshisha University, 1-3 Tatara Miyakodani, Kyotanabe, Kyoto 610-0394, Japan; namiyamoto@mail.doshisha.ac.jp (N.M.); kiyoshi.hirose@komatsu-u.ac.jp (K.H.); 3 Department of Production System Engineering and Sciences, Komatsu University, Nu 1-3 Shicho-machi, Komatsu Ishikawa 923-8511, Japan; 4 Department of Sport and Exercise Science, University of Salzburg, 5020 Salzburg, Austria; thomas.stoeggl@sbg.ac.at; 5 Faculty of Sport and Health Sciences, University of Jyväskylä, FI-40014 Jyväskylä, Finland; vesa.linnamo@jyu.fi; 6 Laboratory of Biomechanics of Skiing, Via Monte Confinale, 10, 23032 Bormio, Italy; steflindinger@gmx.com; 7 Faculty of Health and Sports Science, Doshisha University, 1-3 Tatara Miyakodani, Kyotanabe, Kyoto 610-0394, Japan

**Keywords:** high-precision GNSS, cross-country skiing, skating techniques, sub-technique classification

## Abstract

A comprehensive analysis of cross-country skiing races is a pivotal step in establishing effective training objectives and tactical strategies. This study aimed to develop a method of classifying sub-techniques and analyzing skiing characteristics during cross-country skiing skating style timed races on snow using high-precision kinematic GNSS devices. The study involved attaching GNSS devices to the heads of two athletes during skating style timed races on cross-country ski courses. These devices provided precise positional data and recorded vertical and horizontal head movements and velocity over ground (VOG). Based on these data, sub-techniques were classified by defining waveform patterns for G2, G3, G4, and G6P (G6 with poling action). The validity of the classification was verified by comparing the GNSS data with video analysis, a process that yielded classification accuracies ranging from 95.0% to 98.8% for G2, G3, G4, and G6P. Notably, G4 emerged as the fastest technique, with sub-technique selection varying among skiers and being influenced by skiing velocity and course inclination. The study’s findings have practical implications for athletes and coaches as they demonstrate that high-precision kinematic GNSS devices can accurately classify sub-techniques and detect skiing characteristics during skating style cross-country skiing races, thereby providing valuable insights for training and strategy development.

## 1. Introduction

Cross-country skiing courses are designed to utilize natural terrain, including uphill, downhill, and flat sections, to reflect the athletes’ technical, tactical, and physical abilities [1]. Cross-country skiing has two main competition styles: classical style and skating style. Different gears, known as sub-techniques with distinct movements, are used in each competition style based on the course inclination. There are seven sub-techniques (G1–G7) in the skating style. G1 involves pushing with one ski while simultaneously using the opposite pole and is primarily employed on challenging terrain or when the skier is fatigued. G2 is used on uphill sections and involves one asymmetrical poling action for every two leg movements. G3 is applied on flat to gradual uphill sections and features one poling action and one leg movement. G4 is used on flat sections and involves one symmetrical poling action for every two leg movements. In G5, skiers primarily rely on their legs to propel themselves forward without using their poles; this technique is typically used on flat or slightly descending terrain. G6 is introduced as a curve technique that combines leg strokes with or without poling action. Finally, G7 is utilized in downhill skiing, where the skier adopts a tuck position without using poles or performing leg actions [2,3,4]. Since each sub-technique provides different skiing velocities [3], strategic performance must select the sub-technique that maximizes skiing velocity depending on the course inclination, muscle strength of the upper body, and gliding performance. It has been reported that upper body power and maximal oxygen uptake are necessary for sub-technique selection. Both tend to show superior performance in cross-country skiing in athletes of higher ability, and it is likely that athletes with superior performance in both abilities use faster sub-techniques [5,6,7].

However, what sub-techniques skiers use more frequently throughout a race has yet to be well known. It is practically challenging to video-capture skiers throughout the entire race. Determining the frequency of sub-technique use is an essential resource for determining the relationship between physical fitness, such as upper body power and maximal oxygen uptake, and the technical characteristics of individual athletes, which is also essential for developing training goals and tactics.

In a previous study of race analysis in skating style, Anderson et al. [3] utilized the Global Navigation Satellite System (GNSS) and video cameras to analyze sub-techniques, skiing velocity, cycle time (CT), cycle length (CL), and other relevant parameters during timed races. However, while accurate position data were available from GNSS data, the sub-technique analysis relied on video cameras, making capturing multiple skiers simultaneously throughout the course challenging. Furthermore, the weight of the measurement equipment, 1.64 kg, was a notable problem. Sakurai et al. [8] conducted a sub-technique classification of roller skis using inertial sensors. However, skiing velocity has yet to be clarified in this study. The Naos sensor developed by Archinisis integrates an inertial sensor, a GNSS device, and a barometric pressure sensor in a single compact device, enabling simultaneous data collection of ski technique, skiing velocity, and position information. Analysis has also been conducted using this device [9,10]. However, it has been reported that the GNSS positioning needs to maintain sufficient accuracy, which may cause errors in position information [11], and the accuracy of the measurements is not sure. These challenges highlight the need for a more effective and accurate method, which we aim to address in this study.

On the other hand, Takeda et al. [12] used a high-precision kinematic GNSS device to measure GNSS position information with extremely high precision to evaluate sub-technique classification and skiing velocity during a classical style timed race for the world’s top-level male skier. In this study, the GNSS device was attached to the head, and technique classification was attempted based on the hypothesis that the vertical movement of the head is different for each sub-technique. They reported that they could classify the sub-techniques (double poling, diagonal stride, kick double polling, and herringbone) with 98% accuracy. Øyvind Gløersen et al. [13] used the same high-precision kinematic GNSS device and classified sub-techniques in skating style techniques based on vertical and horizontal head movement features. They reported correctly classifying sub-techniques G2, G3, and G4 with an accuracy of 92.1% to 97.1%. However, the study by Øyvind Gløersen et al. [13] attempted classification during roller skiing on paved trails. To the authors’ knowledge, no research has been done on the sub-technique classification of actual skating style on unstable snow surfaces. Since the head movement in the skating style does not differ much from one sub-technique to another compared with the classical style, it is crucial to capture head movements in detail and with high accuracy.

The position information measurement technology of GNSS devices has been improving at an accelerated pace in recent years. Accordingly, momentum and running speed analyses have been conducted using GNSS devices in many sports, such as soccer and rugby. Most of these GNSS devices use relative positioning GPS (differential GPS), which has a significant measurement error of about 1 m, and the measurement frequency is generally 10 Hz [14]. Sports activities are high speed, and to detect slight differences in skiing velocity and position information, it is necessary to measure position information with high accuracy and to have a high measurement frequency. This point was also pointed out by Takeda et al. [12] in another study of the cross-country skiing sub-technique. Miyamoto et al. developed a compact, lightweight, low power consumption, and 10 Hz update rate post-processing kinematic (PPK) GNSS logger (AT-H-02, AOBA Technologia LLC, Sendai, Japan) that is suitable for skiing measurements [15]. The AT-H-02 does not perform the positioning calculations for navigation commonly performed by GNSS devices, but it specializes in logging the raw data required for PPK [15]. Kinematic positioning based on carrier phase measurements provides higher accuracy than differential GPS, reaching sub-centimeters. Furthermore, a GNSS logger (simpleRTK3B Pro, ArduSimple, Lleida, Spain) has been developed, which maintains its size, weight, and measurement accuracy while significantly increasing the sampling frequency from the traditional 10 Hz to 100 Hz. This advancement of GNSS technologies enhances temporal resolution and provides unprecedented precision in data acquisition, enabling more accurate classification of sub-techniques in skating style performed on unstable snow surfaces. We hypothesized that this high-precision GNSS device could accurately classify sub-techniques in cross-country ski skating style. Based on this, the present study used a high-precision kinematic GNSS device to classify sub-techniques and analysis of skiing characteristics in cross-country ski skating style.

## 2. Materials and Methods

### 2.1. Overall Design

**Figure 1:**
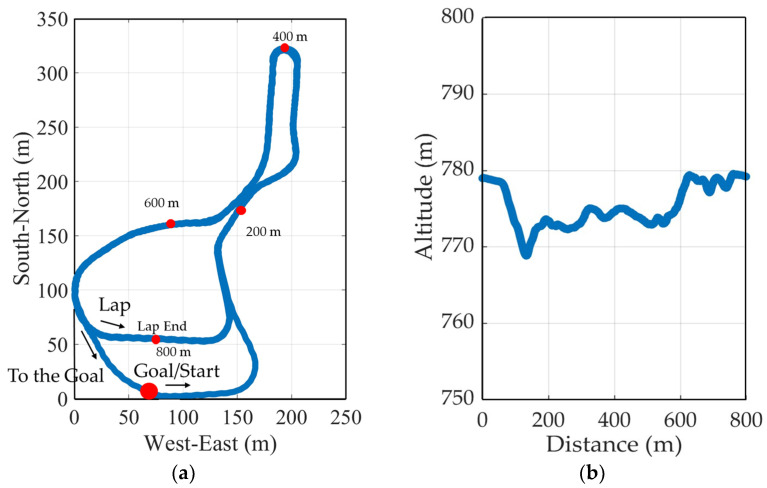
The figure shows the Ikenotaira cross-country ski course, Japan, used in this study. The plotted data were obtained from the study subject, covering one lap of 0.8 km. The figure shows the course profile’s plan view data (**a**) and course inclination data (**b**).

**Figure 2:**
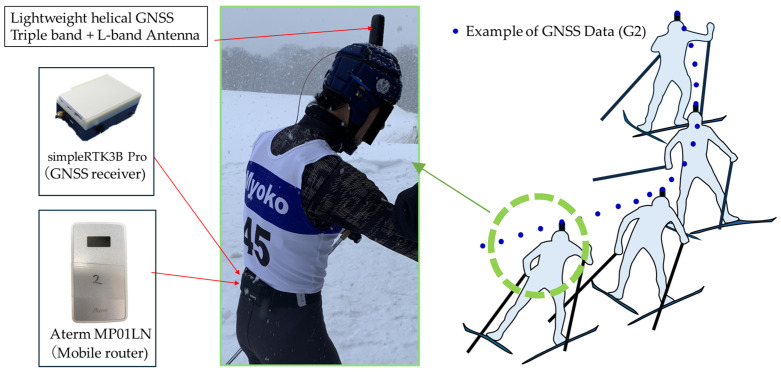
This picture and image show the experimental setup. The GNSS antenna was attached to the skier’s head, and the receiver and mobile router were stored in a small bag at the skier’s waist. This setup obtained head positioning data (latitude, longitude, altitude, and VOG) during the timed race.

Two male athletes (subject A and subject B) participated in a skating style timed race on the Ikenotaira cross-country ski course in Japan, which was 4.1 km long (five laps, with the first lap being 0.9 km and the second to fifth laps being 0.8 km each; Figure 1). Subject A was a former member of the Japanese national team, and subject B was an elite athlete in the Japanese U-15 category. The subjects used their own skis, poles, and boots. A GNSS device was attached to the subjects to obtain head position data during the timed race. The GNSS device consisted of an antenna (lightweight helical GNSS triple band + L-band antenna, ArduSimple, Lleida, Spain; size, 40 mm φ × H82.8 mm; weight, 25 g) and a receiver (simpleRTK3B Pro, ArduSimple, Lleida, Spain; size, W59 mm × D87 mm × H33 mm; weight, 137 g). The antenna was attached to the subjects’ heads, and the receiver was stored in a small bag at their waist (Figure 2). The receiver was equipped with a GNSS module Mosaic-X5 (Septentrio, Leuven, Belgium), capable of performing RTK positioning at a maximum sampling frequency of 100 Hz. The positioning accuracy was 0.6 cm + 0.5 ppm horizontally and 1 cm + 1 ppm vertically. To enable the receiver to communicate with the base station (NTRIP Caster/NTRIP Server) and obtain correction information (RTCM), a mobile router (Aterm MP01LN, NEC, Tokyo, Japan; size, W50 × H12 × D91 mm; weight, 71 g) was stored in the small bag along with the receiver. During the timed race, the subjects were followed by a snowmobile from behind, and all sub-techniques were recorded throughout the entire race using a video camera (Hero9, GoPro, San Mateo, California, USA). This study was conducted with the approval of the Ethics Committee of Doshisha University (No. 23042).

### 2.2. Data Processing

NMEA messages obtained the trajectory of head movement relative to the latitude, longitude, altitude, and VOG during the timed race, and the data analysis was conducted using MATLAB 2024a.

The movement of the head obtained from GNSS data included the influence of the course inclination and curves; therefore, removing these effects allowed for the trajectory of pure head movement. The course inclination was derived by calculating a moving average from the altitude data. We used a sliding window approach with a window size of 2k + 1 data points centered on each data point (where $\left(i\right)$ represents a specific data point). In this study, k was set to 55 (1.1 s). The course inclination is given by

(1) $$\mathrm{Course}\;\mathrm{Inclination}\left(i\right)=\frac{1}{2k+1}\sum_{j=i-k}^{i+k}\mathrm{alt}\left(j\right)$$

With this procedure, it was possible to draw the course inclination for the entire course, as shown in Figure 1b. The change in the trajectory of the net vertical movement of the head was extracted by subtracting the course inclination data from the altitude data obtained from GNSS [12].

When attempting to remove the effects of course curves using a similar method with latitude and longitude data, the influence of the subject’s horizontal movements was reflected, making it challenging to smoothly represent the changes in course curves. Therefore, the calculation was performed using the following procedure: First, the LLH (latitude, longitude, altitude) coordinates were converted into the ENU (east-north-up) coordinates ($v_{i}\left(1\right),v_{i}\left(2\right),v_{i}\left(3\right)$) using the given latitude, longitude, and altitude with the MATLAB function llh2enu. Here, $v_{i}\left(1\right)$ represents the eastward component, $v_{i}\left(2\right)$ represents the northward component, and $v_{i}\left(3\right)$ represents the upward component. Next, the horizontal components of the ENU coordinates ($v_{i}\left(1\right),v_{i}\left(2\right)$) were converted into polar coordinates ($\theta_{\mathrm{GNSS}}\left(i\right),\;\rho \left(i\right)$) by Equation (2). Like Equation (1), the moving average of $\theta_{\mathrm{GNSS}}$ was then computed to derive $\theta_{ave}$ by Equation (3). In this study, m was set to 52 (1.04 s). By subtracting$\;\theta_{\mathrm{ave}}\left(i\right)$ from $\theta_{\mathrm{GNSS}}\left(i\right)$, the net angle change $\theta_{\mathrm{net}}\left(i\right)$ was obtained by Equation (4). Finally, $\theta_{\mathrm{net}}$ was used to compute the horizontal movement of the head by Equation (5).

(2) $$\theta_{\mathrm{GNSS}}\left(i\right)=\mathrm{atan}2\left(v_{i}\left(2\right),v_{i}\left(1\right)\right)\;\rho \left(i\right)=\sqrt{v_{i}{\left(1\right)}^{2}+v_{i}{\left(2\right)}^{2}}$$

(3) $$\theta_{\mathrm{ave}}\left(i\right)=\frac{1}{2m+1}\sum_{j=i-m}^{i+m}\theta_{\mathrm{GNSS}}\;\;\left(j\right)$$

(4) $$\theta_{\mathrm{net}}\left(i\right)=\theta_{\mathrm{GNSS}}\left(i\right)-\theta_{\mathrm{ave}}\left(i\right)$$

(5) $$\mathrm{Net}\;\mathrm{horizontal}\;\mathrm{movement}\left(i+1\right)=\mathrm{Net}\;\mathrm{horizontal}\;\mathrm{movement}\left(i\right)+\rho \left(i\right)\cdot \sin\left(\theta_{\mathrm{net}}\left(i\right)\right)$$

The windows sizes of 1.10 s and 1.04 s used for calculating the moving average in Equations (1) and (3), respectively, were not derived from a specific dataset but were instead determined as the values that best reflected the changes in the slope inclination and curve radius throughout the entire race course in this study. Therefore, it is possible that other values may be more appropriate for courses other than those used in this study.

### 2.3. Sub-Technique Classification

In order to facilitate the classification of sub-techniques (G1–G7), techniques involving poling action were the primary focus of the analysis. This was because techniques with poling action exhibited noticeable vertical and horizontal head movements, making it easier to capture key points for classification. As a result, four techniques involving poling action—G2, G3, G4, and G6P (we will refer to G6 with poling action as G6P, without poling action as G6N, and G6 as a general term encompassing both poling action and non-poling action)—were included in the analysis. Additionally, movements involving poling that could not be clearly classified (e.g., moments of loss of balance or double poling) were categorized as “others”. G1 was not used at all. G5, G6N, and G7 were techniques that did not involve poling action. In the case of G5 and G6N, it was difficult to determine whether the movements were due to a loss of balance or a change in direction. Furthermore, G5 and G6N were used in downhill sections and had low usage frequency. Along with G7, which was a technique used in downhill sections, G5, G6N, and G7 were categorized together as “downhill”. First, one poling action was defined as one cycle in each sub-technique. One poling action was extracted by classifying peaks in the net vertical movement of the head waveform. Next, the typical waveform patterns for each sub-technique were defined based on the differences in the patterns of net vertical and horizontal head movements and VOG changes within one cycle. The differences in waveform patterns were focused on the shape, amplitude, timing, and frequency of peaks and valleys. Finally, the sub-techniques during the timed race were manually (visually) classified based on the typical waveform patterns.

The validity was verified by comparing the data classified visually from video data obtained from a video camera mounted on the snowmobile with the data classified using GNSS data. Experts with over ten years of skiing experience, different from those who classified the techniques based on waveform patterns, used Kinovea video software (ver. 2023.1.2) to classify the techniques from the video data. The data classified from the video were used as the validity standard, and the consistency with the data classified from the GNSS was verified. The match rate (%) was calculated for all techniques and sub-techniques (%Match = GNSS data/Video data).

### 2.4. Analysis of Skiing Characteristics

The usage ratio of each sub-technique concerning time and distance during the timed race was calculated. Based on the head position data, the straight-line distance moved by the head during one cycle was calculated as CL, and the time required for one cycle was calculated as CT. Furthermore, the skiing velocity was calculated by dividing CL by CT. The course inclination at which each sub-technique was used was calculated based on the difference in course incline data between one cycle’s start and end points relative to CL. Using Excel statistics, a one-way analysis of variance (ANOVA) was conducted on CT, CL, skiing velocity, and course inclination for each of the two subjects. Bonferroni multiple comparisons were performed if significant variance was observed to examine the differences between the sub-techniques. The significance level was set to alpha = 0.05.

## 3. Results

### 3.1. The Typical Waveform Pattern of Each Sub-Technique

**Figure 3:**
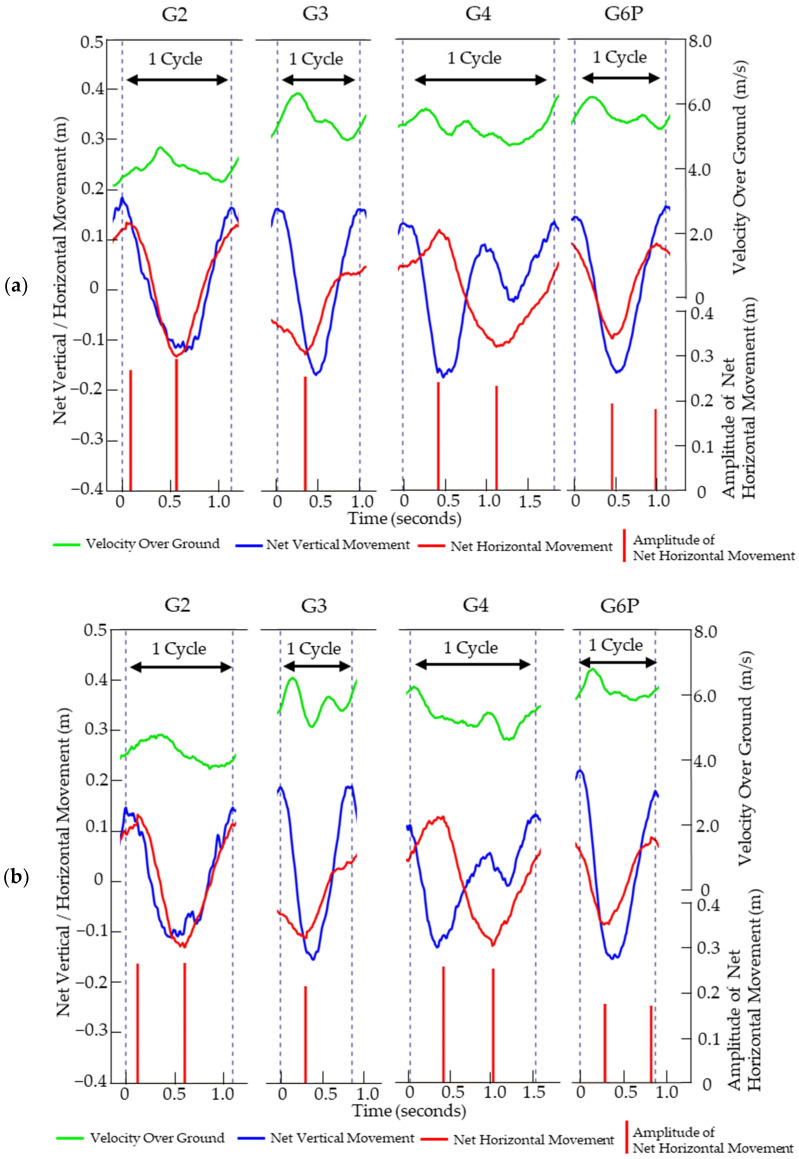
The figure shows the typical waveform patterns of subject A (**a**) and subject B (**b**) for G2, G3, G4, and G6P. The black dashed lines indicate the points where the net vertical head movement reaches a peak. The interval between two black lines represents one cycle. The green lines indicate the VOG. The blue waveform shows the trajectory of the net vertical head movement. The red waveform shows the trajectory of the net horizontal head movement. The red bars indicate the amplitude of the net horizontal head movement.

The typical waveform patterns for each sub-technique were defined based on the differences in the patterns of net vertical and horizontal head movements and changes in VOG within one cycle as follows (Figure 3). G3 was characterized by a single peak or valley in the net horizontal head movement waveform within one cycle. G2 was characterized by one peak and one valley in the net horizontal head movement waveform within one cycle. Like G2, G4 had one peak and one valley in the net horizontal head movement waveform within one cycle. However, G4 was characterized by a large wave followed by a slight wave in the net vertical head movement waveform within one cycle. G6P had the same waveform as G2 in net vertical and horizontal head movements. However, G2 and G6P could be distinguished by the following criteria. Compared with G2, G6P had a smaller amplitude in net horizontal head movement. Additionally, G6P had a higher VOG. The timing of the peaks or valleys in net horizontal head movement also differed. In G2, these were observed in the first half and middle of the cycle, whereas in G6P, they appeared in the middle and latter half. These typical waveform patterns for each sub-technique were observed in both subjects.

### 3.2. Validity of Sub-Technique Classification Based on Waveform Patterns

**Figure 4:**
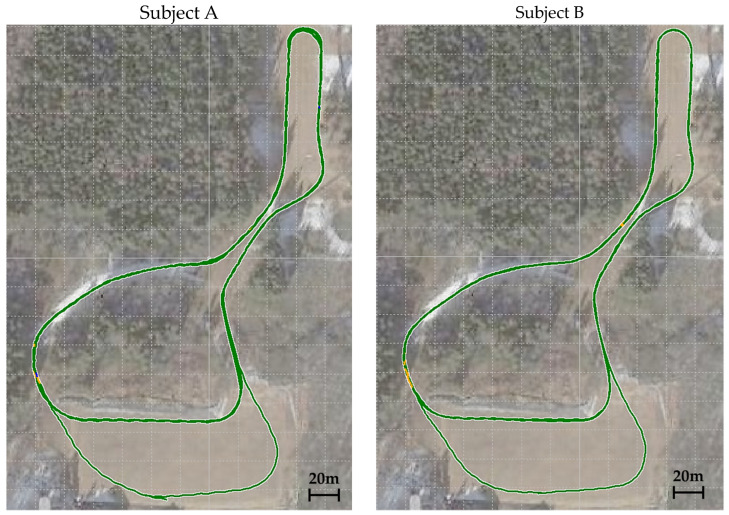
The figure shows the quality of the positional data obtained from the RTK GNSS devices for subject A and subject B. The green color indicates the fix solution, the orange color indicates the float solution, and the blue color indicates the dGNSS solution.

The fix rate of the data obtained from the GNSS device was 99.1% for subject A and 98.6% for subject B (Figure 4).

The sub-techniques were classified with high accuracy for both subjects. The match rates of sub-technique classifications from video data and GNSS data are shown in Table 1 and Table 2. Here, we also investigated the effects of different GNSS solutions used, such as fix, float, and dGNSS, for sub-technique classification. The match rates obtained using both fix-only solutions and all GNSS solutions including fix, float, and dGNSS were as follows: For subject A, the match rates using fix solutions were 97.4% for G2, 98.5% for G3, 98.1% for G4, and 97.0% for G6P, resulting in an overall match rate of 97.6% for G2, G3, G4, and G6P. When using all GNSS solutions, the match rates were 97.4% for G2, 98.5% for G3, 98.2% for G4, and 97.0% for G6P, resulting in an overall match rate of 97.7% (Table 1). For subject B, the match rates using fix solutions were 96.3% for G2, 98.9% for G3, 94.7% for G4, and 100% for G6P, with an overall match rate of 96.6% for G2, G3, G4, and G6P. When using all GNSS solutions, the match rates were 95.0% for G2, 98.6% for G3, 95.1% for G4, and 98.8% for G6P, resulting in an overall match rate of 96.0% (Table 2).

### 3.3. Characteristics of Each Sub-Technique

**Figure 5:**
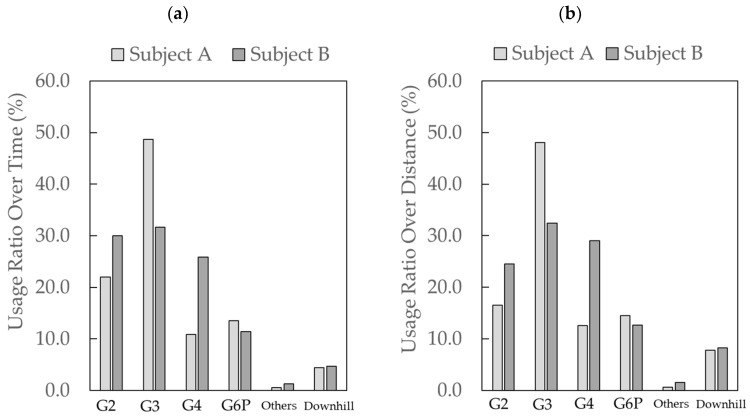
This figure shows the usage ratio over time (**a**) and the ratio over distance (**b**) for each sub-technique during the timed race.

The characteristics of each sub-technique were analyzed using all GNSS solutions. The time required for the timed race was 909 s for subject A and 860 s for subject B. The time percentages for each technique were as follows: G2 was 22.0% and 30.0%, G3 was 48.7% and 31.6%, G4 was 10.9% and 25.8%, G6P was 13.5% and 11.4%, “others” was 0.5% and 1.3%, and downhill was 4.4% and 4.7% (Figure 5a). The distance percentages for each technique were as follows: G2 was 16.5% and 24.5%, G3 was 48.1% and 32.4%, G4 was 12.6% and 29.0%, G6P was 14.5% and 12.6%, “others” was 0.6% and 1.5%, and downhill was 7.7% and 8.2% (Figure 5b).

**Figure 6:**
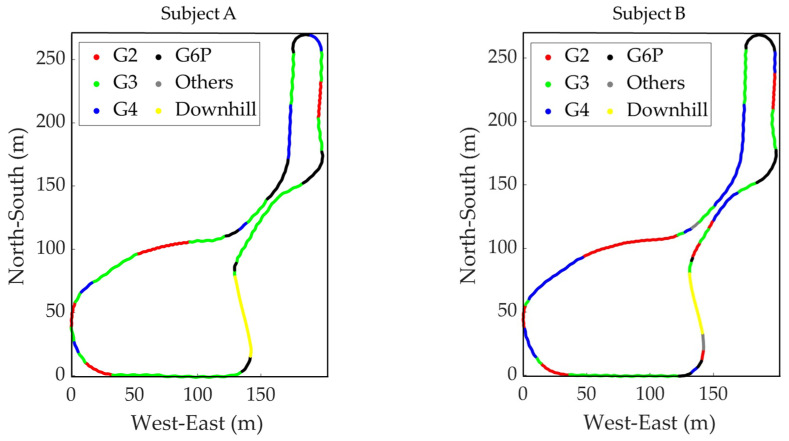
The distribution of sub-techniques used by two subjects during the second lap of the timed race is shown on the course profile’s plan view data.

**Figure 7:**
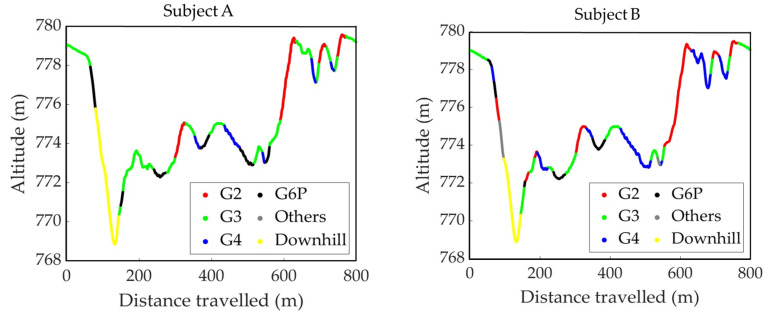
The course inclination data show the distribution of sub-techniques used by two subjects during the second lap of the timed race.

**Figure 8:**
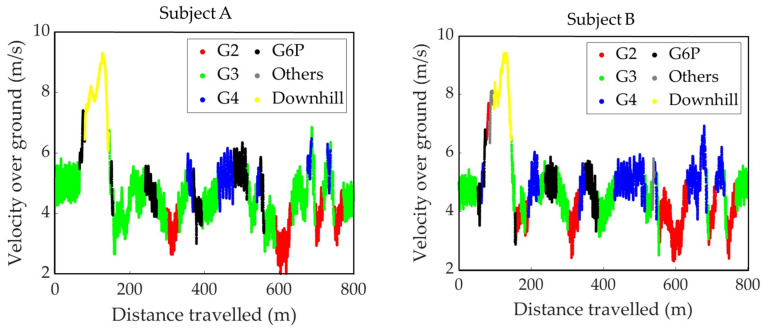
The distribution of sub-techniques used by two subjects during the second lap of the timed race. The *X*-axis indicates the distance traveled, and the *Y*-axis indicates the VOG of the skier’s head.

The sub-techniques used during the second lap of the timed race are shown on the course profile’s plan view (Figure 6) and the course inclination (Figure 7). Figure 8 is the VOG of the skier’s head plotted on the vertical axis.

**Figure 9:**
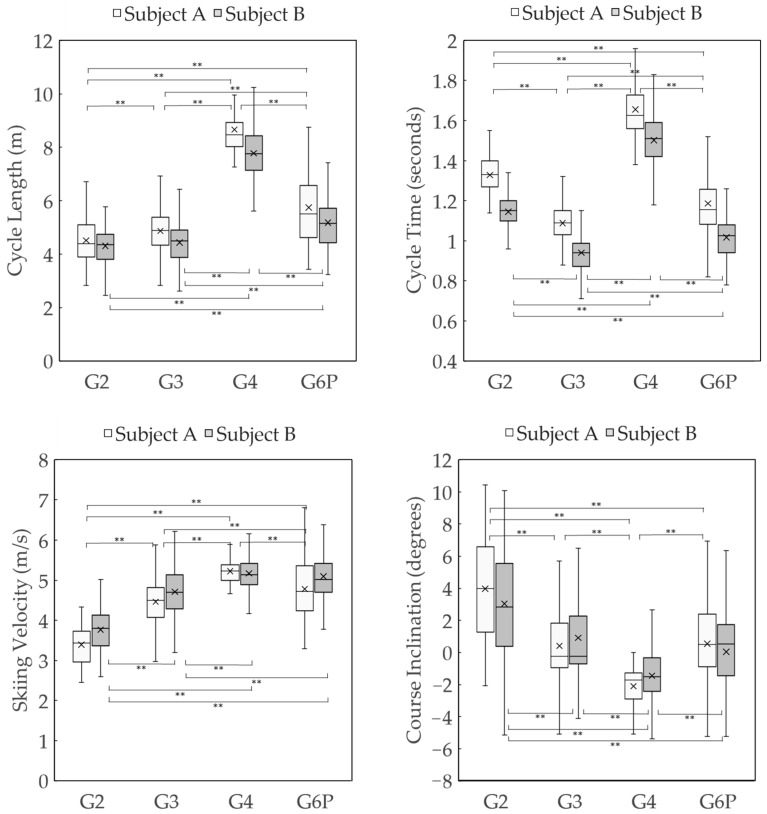
This figure shows the CL, CT, skiing velocity, and course inclination data for subjects A and B’s sub-techniques during the timed race. Each sub-technique cycle was defined from the vertical movement peak at the waveform data’s head to the next peak. The horizontal line within each box represents the median value of the dataset, while the “x” symbol denotes the mean value. ** indicates a significance level of *p* < 0.01.

The average CLs for each sub-technique during the timed race for subjects A and B were as follows (Figure 9): G2 was 4.52 ± 0.82 m and 4.31 ± 0.75 m, G3 was 4.87 ± 0.83 m and 4.44 ± 0.85 m, G4 was 8.66 ± 1.23 m and 7.78 ± 1.16 m, and G6P was 5.75 ± 1.74 m and 5.18 ± 0.98 m. The average CTs were as follows: G2 was 1.33 ± 0.10 s and 1.14 ± 0.09 s, G3 was 1.09 ± 0.11 s and 0.94 ± 0.11 s, G4 was 1.66 ± 0.18 s and 1.50 ± 0.16 s, and G6P was 1.19 ± 0.18 s and 1.02 ± 0.12 s. The average skiing velocities were as follows: G2 was 3.39 ± 0.50 m/s and 3.77 ± 0.57 m/s, G3 was 4.46 ± 0.55 m/s and 4.71 ± 0.64 m/s, G4 was 5.22 ± 0.32 m/s and 5.17 ± 0.51 m/s, and G6P was 4.78 ± 0.79 m/s and 5.10 ± 0.82 m/s. The average course inclination for each technique was as follows: G2 was 3.98 ± 3.24 degrees and 3.04 ± 3.42 degrees, G3 was 0.40 ± 2.66 degrees and 0.91 ± 2.79 degrees, G4 was −2.10 ± 1.45 degrees and −1.45 ± 2.31 degrees, and G6P was 0.55 ± 3.80 degrees and 0.04 ± 3.98 degrees. When comparing the CL, CT, skiing velocity, and course inclination for each sub-technique between subjects A and B, subject A showed significant differences in all sub-techniques except for the course inclination of G3 and G6P. Subject B showed significant differences in all sub-techniques except for the CL of G2 and G3, the skiing velocity of G4 and G6P, and the course inclination of G3 and G6P.

**Figure 10:**
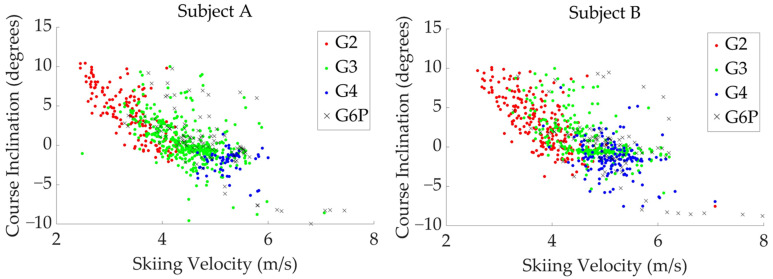
The distribution of four sub-techniques used by two subjects during the timed race is shown with skiing velocity (*X*-axis) and course inclination (*Y*-axis).

**Figure 11:**
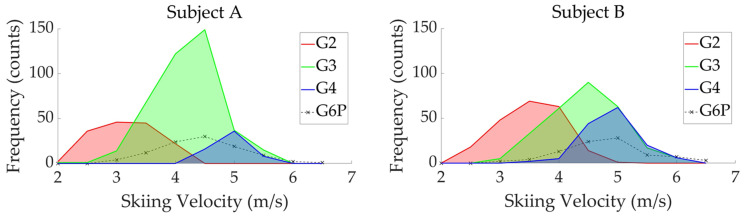
The distribution of four sub-techniques used by two subjects during the timed race is shown as a histogram of skiing velocity frequencies.

**Figure 12:**
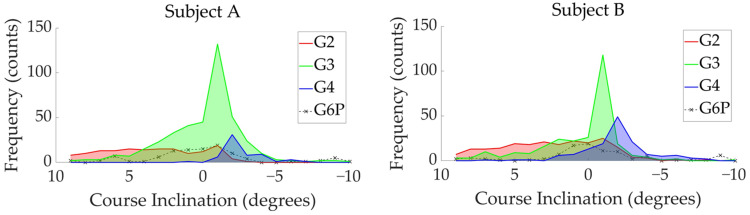
The distribution of four sub-techniques used by two subjects during the timed race is shown as a histogram of course inclination frequencies.

The distribution of four sub-techniques used by two subjects during the timed race is shown in Figure 10, Figure 11 and Figure 12. Figure 10 shows the sub-technique distribution plotted against skiing velocity (*X*-axis) and course inclination (*Y*-axis). Figure 11 and Figure 12 show the frequency distribution of sub-techniques as histograms for skiing velocity (Figure 11) and course inclination (Figure 12), respectively. Each sub-technique was classified and described based on GNSS data from the skier’s head.

## 4. Discussion

This study aimed to establish a method for analyzing skating techniques on snow using a high-precision kinematic GNSS device. Based on the head movement patterns exhibited by each sub-technique, the classification accuracy was 95.0–98.6% for G2–G4 based on all GNSS solutions in this study. Øyvind Gløersen et al. [13] achieved 92.1–97.1% accuracy for G2–G4 in classifying sub-techniques during roller skiing. The results of our study indicated that high-accuracy classification is achievable on snow, as well as in previous studies. For G6, our study achieved an accuracy of over 97.0%, whereas the preliminary study reported an accuracy of 88%. While the preliminary study classified G6 based on changes in skiing direction, we specifically focused on G6 that included poling actions. Our findings demonstrated that turns involving poling actions could be accurately classified. The waveforms of vertical and horizontal head movements and VOG derived from head position data obtained from the GNSS device exhibited characteristic patterns for each sub-technique. The waveform from one peak to the next in the vertical movement represented one poling action. Peaks or valleys in the horizontal movement waveforms represented direction changes and indicate leg action. The GNSS data waveforms showed that the relationship between the number of poling actions and leg actions per cycle corresponded accurately with the movements of each sub-technique demonstrated in previous studies [2,3]. For sub-techniques G2, G4, and G6P, the relationship between the number of poling actions and leg actions within one cycle was the same. However, it was possible to classify these sub-techniques based on the timing differences of the peaks in net horizontal head movements, waveform, VOG changes, and differences in the amplitude of net horizontal movements. The waveforms of G2 and G6P were very similar. G2 was reflected in VOG as a slower technique because it tended to be used in areas with steeper inclines [3,16]. The differences in the timing of horizontal movement peaks and amplitude may be influenced by G6P being a sub-technique used while turning on the course. Regarding the classification of G6, various criteria have been used, including classification based on the displacement direction (≤10°) [13], including it in skating without a pole [16], and including it in G2 [8]. Research focusing on G6 has mainly addressed course inclination less than 0° [17], and studies on its use in other types of terrain are still lacking. Our study revealed that G2 and G6P were sub-techniques with distinct skiing characteristics, differing in skiing velocity, CL, CT, and course inclination. Detailed analysis of G6 is also necessary to thoroughly analyze the skiing characteristics of athletes in races.

The GNSS data also revealed skiing characteristics for each sub-technique. The results showed that the ratio of each used sub-technique varied between the two subjects depending on time and distance. This suggested that the sub-techniques may differ depending on the skier’s fitness and skill, even on the same course. Of the main sub-techniques for skating (G2, G3, and G4), G4 was the fastest for both subjects, followed by G3 and G2. The fact that G4 was used on the course with the most minor slope and G2 on the course with the steepest slope suggested that the course’s slope influenced the choice of sub-technique. The primary purpose of this study was to use GNSS for technique discrimination of skating technique and not to compare the technique characteristics of high-performing and non-performing athletes. However, we would like to add a few considerations to show that this study can also classify the sub-technique use ratio and technique characteristics of skiers with different skiing performances. Subject A was a former representative of Japan’s national cross-country skiing team and continued training and competing in national competitions after retiring from the national team. On the other hand, subject B was a 15-year-old junior high school athlete, suggesting that subject A had far greater physical strength and technique. Namely, as shown in Figure 10, Figure 11 and Figure 12, subject A used G3, which required upper body strength at high speeds, even on steep slopes. This indicates that the higher-performing skier may be able to use faster techniques on the same course incline compared with lower-performing skiers. Also, as shown in Figure 7, subject A used the same technique more consistently than subject B, depending on the course’s slope. In contrast, subject B used multiple techniques even at the same slope, indicating that the skiers with superior performance have a higher ability to use the most appropriate technique for a given situation consistently. However, the results of the time race conducted in this study showed that subject A was 49 s slower than subject B. During the time race of subject A, there was heavy snow in the second half of the race, and the ski gliding performance was inferior from the middle of the race. In addition, since the course used in this study had a relatively gentle slope, it is undeniable that the downhill skiing performance also significantly impacted the performance times. This resulted in the time of subject A, who was supposed to have high performance, being slower than that of subject B. This should be understood as a difficulty in experiments on snow. Ideally, such an experiment should be conducted on an occasion when weather conditions are good. In any case, the authors would like to emphasize that the analysis method of this study will provide concrete suggestions for the performance and technical analysis of skiers during races.

In this study, we achieved a high-accuracy classification of skating sub-techniques and analyzed skiing characteristics on snow using a high-precision GNSS device. The strength of the high-precision GNSS device lies in its ability to provide detailed data on skiing characteristics (CL, CT, skiing velocity, course inclination, and sub-technique selection) to athletes and coaches. Knowing their skiing characteristics can benefit coaches and athletes when planning race tactics. Furthermore, elucidating the relationship between these skiing characteristics and physical fitness metrics, such as upper body power and maximal oxygen uptake, can contribute to developing training goals.

However, several limitations were identified in our study. First, head position data alone cannot directly measure body rotation or center of gravity load, as can be done with sub-technique analysis based on IMU sensors [18,19], nor can it provide detailed motion analysis of body parts as achieved with three-dimensional video analysis. Combining the analysis of the differences in head movements revealed in this study with IMU sensors and three-dimensional video analysis may allow for a more detailed examination of the athletes’ technique analysis. Second, the course used in this study is an open area also used as a golf course with a very high GNSS fix rate (>99.1% and >98.6%). However, obtaining fixed solutions in places where the course was narrow and with tall trees on both sides was challenging. Even in such areas, we captured the main classification features with float solutions and achieved high accuracy. On the other hand, on courses with many obstacles, such as trees and buildings, the fixed rate may decrease, resulting in lower classification accuracy. To improve the validity of this method further, it is essential to demonstrate it on different terrains and courses. To maintain accuracy, it may be necessary to integrate IMU data or use methods to optimize position information [20] to compensate for areas where fixed solutions are not obtained. Finally, this study had a small sample size of only two subjects, and sub-technique classification relied on visual observation. In the future, to extend this classification method, increasing the number of subjects and automating the process through the development of classification algorithms will be significant research challenges. Furthermore, if our analysis methods can be used to analyze the day’s training, races, and even training effectiveness, coaches and athletes will have more detailed insight into analyzing athletes’ techniques and determining training effectiveness.

## 5. Conclusions

Based on the results of this study, it was demonstrated that by attaching a high-precision kinematic GNSS device to the skier’s head during a cross-country ski skating style timed race on snow, sub-techniques could be classified based on the vertical and horizontal head movements, as well as differences in VOG. Additionally, it was shown that skiing characteristics (CL, CT, skiing velocity, and course inclination) could be derived from the GNSS data.

## Figures and Tables

**Table 1:** This table shows the consistency between the classifications using video data and GNSS data, based on both fix-only solutions and all GNSS solutions, including fix, float, and dGNSS for subject A.

Fix-Only Solutions	GNSS Classification								
		G2	G3	G4	G6P	Others	None	Total	Accuracy (%)
Video classification	G2	148	0	0	4	0	0	152	97.4
	G3	1	387	1	3	0	1	393	98.5
	G4	0	1	52	0	0	0	53	98.1
	G6P	0	2	1	97	0	0	100	97.0
	Others	0	0	2	0	1	0	3	33.3
	None	0	0	0	0	1		1	
	Total	149	390	56	104	2	1		
**All Solutions**	**GNSS Classification**								
		**G2**	**G3**	**G4**	**G6P**	**Others**	**None**	**Total**	**Accuracy (%)**
Video classification	G2	150	0	0	4	0	0	154	97.4
	G3	1	405	1	3	0	1	411	98.5
	G4	0	1	56	0	0	0	57	98.2
	G6P	0	2	1	97	0	0	100	97.0
	Others	0	0	2	0	1	0	3	33.3
	None	0	0	0	0	1		1	
	Total	151	408	60	104	2	1		

**Table 2:** This table shows the consistency between the classifications using video data and GNSS data, based on both fix-only solutions and all GNSS solutions, including fix, float, and dGNSS for subject B.

Fix-Only Solutions	GNSS Classification								
		G2	G3	G4	G6P	Others	None	Total	Accuracy (%)
Video classification	G2	207	0	2	6	0	0	215	96.3
	G3	1	268	0	2	0	0	271	98.9
	G4	1	0	124	0	6	0	131	94.7
	G6P	0	0	0	81	0	0	81	100
	Others	0	1	1	0	2	0	4	50.0
	None	2	0	0	2	0		4	
	Total	211	269	127	91	8	0		
**All Solutions**	**GNSS Classification**								
		**G2**	**G3**	**G4**	**G6P**	**Others**	**None**	**Total**	**Accuracy (%)**
Video classification	G2	211	0	5	6	0	0	222	95.0
	G3	1	274	0	3	0	0	278	98.6
	G4	1	0	135	0	6	0	142	95.1
	G6P	0	1	0	81	0	0	82	98.8
	Others	0	1	1	0	2	0	4	50.0
	None	2	0	0	2	0		4	
	Total	215	276	141	92	8	2		

## Data Availability

The datasets generated during and analyzed during the current study are available from the corresponding author upon reasonable request.
